# Editor *ad interim* – prospects and reality

**DOI:** 10.3325/cmj.2018.59.288

**Published:** 2018-12

**Authors:** Anton Glasnović

**Affiliations:** Interim Editor-in-Chief, *Croatian Medical Journal*, Zagreb, Croatia *anton.glasnovic@cmj.hr*

By assuming the role of the editor in chief of the *Croatian Medical Journal* (*CMJ*), I took over the task of maintaining the reputation of a journal, which although it is one of the best general medical journals in an EU member country, is still a small medical journal at the scientific periphery. It is difficult for such a journal to compete with the likes of the *Lancet, British Medical Journal (BMJ), or Journal of American Medical Association* (*JAMA*), although we try to uphold the same high standards of reporting research and publication integrity. By working in the *CMJ* as a full-time employee for two years I have become familiar with all the aspects of journal publishing process, and therefore I know that we should be realistic. The *CMJ*'s goal from the beginning has not been to publish the most influential studies, but to allow scientists who have good ideas but come from countries with lower investment in science to publish their work in an internationally indexed journal. And even more than that, our goal has been to teach younger, but also more experienced scientists, how to set up research well, write good scientific articles, and give them a springboard for later publication in more prestigious journals (1-3). Therefore, our greatest profit is to see that Croatian scientists, as well as scientists from similar countries, after publishing in the *CMJ*, continued their series of quality publications in other well-known journals from their field of interest (1-3).

However, despite the author-helpful policy and diamond open access, maintaining the citation impact indicators and attracting quality manuscripts remain to be challenging tasks. There are many factors affecting the authors' choice of a journal (4,5). Although we cannot increase the impact factor or journal prestige overnight, we must begin by first tackling the issues that we can change quickly. One of these aspects is the simplicity of manuscript upload system. In the next period, we will make it even easier and quicker for both the authors and reviewers to upload their submissions, because the simplicity of the manuscript upload can sometimes be crucial in author's decision whether to submit a manuscript. Another very important factor in author's journal selection is manuscript turnover time. A considerable proportion of manuscript turnover time is spent on peer review process, the duration of which cannot entirely be influenced by editors. However, it is completely in our power to shorten the submission-to-first-decision time. Previous editors have managed to shorten this interval, but I will further strive to optimize the journal workflow and enable the authors to receive the first response about the submitted manuscript in a very short time-frame.

In the next year, we will continue publishing Knowledge Landscapes, a series of texts on the challenges the digital society presents to medicine, which has been a standard column for the past two years. We also plan a new column about biostatistics. This column will deal with new statistical concepts and dilemmas that researchers face when setting up research, and will help them apply biostatistical knowledge in their work. Also, we will try to add new thematic issues to the standard ones, such as International Society for Applied Biological Sciences thematic issue scheduled for June 2019.

Finally, it will be hard to surpass the excellent previous editors in chief, but I see the editor’s job to be honest and rewarding, so I am sure when I leave the journal it will be ranked even higher and be more influential. The main goal in next period will be to increase the journals' impact factor and to attract more quality articles, both from Croatian and foreign scientists. I hope that in achieving this goal, I will have your help.
